# A New Application of Internet of Things and Cloud Services in Analytical Chemistry: Determination of Bicarbonate in Water

**DOI:** 10.3390/s19245528

**Published:** 2019-12-14

**Authors:** J. V. Capella, Alberto Bonastre, Rafael Ors, Miguel Peris

**Affiliations:** 1 Instituto ITACA, Universitat Politècnica de València, Ciutat Politècnica de la Innovació, Camino de Vera s/n, 46022 Valencia, Spain; jcapella@itaca.upv.es (J.V.C.); rors@itaca.upv.es (R.O.); 2 Department of Chemistry, Universitat Politècnica de València, Camino de Vera s/n, 46022 Valencia, Spain

**Keywords:** smart sensors, cloud services, Internet of Things, bicarbonate, water analysis

## Abstract

In a constantly evolving world, new technologies such as Internet of Things (IoT) and cloud-based services offer great opportunities in many fields. In this paper we propose a new approach to the development of smart sensors using IoT and cloud computing, which open new interesting possibilities in analytical chemistry. According to IoT philosophy, these new sensors are able to integrate the generated data on the existing IoT platforms, so that information may be used whenever needed. Furthermore, the utilization of these technologies permits one to obtain sensors with significantly enhanced features using the information available in the cloud. To validate our new approach, a bicarbonate IoT-based smart sensor has been developed. A classical CO_2_ ion selective electrode (ISE) utilizes the pH information retrieved from the cloud and then provides an indirect measurement of bicarbonate concentration, which is offered to the cloud. The experimental data obtained are compared to those yielded by three other classical ISEs, with satisfactory results being achieved in most instances. Additionally, this methodology leads to lower-consumption, low-cost bicarbonate sensors capable of being employed within an IoT application, for instance in the continuous monitoring of HCO_3_^−^ in rivers. Most importantly, this innovative application field of IoT and cloud approaches can be clearly perceived as an indicator for future developments over the short-term.

## 1. Introduction

Carbon dioxide (CO_2_) emissions, mainly due to fossil fuel combustion, land use change, and other sources, have dramatically increased over the past one hundred years. In this sense, natural waters, including oceans, lakes, and streams, serve as the major sinks for increasing levels of CO_2_ in the atmosphere. The net flux of this gas into these water reservoirs is primarily driven by solubilization of carbon dioxide. Its dissociation mainly generates bicarbonate (HCO_3_^−^) and carbonate (CO_3_^2−^) ions, which together constitute the total carbonates present. They represent a major source of alkalinity and buffering in natural aqueous environments [1]. Bicarbonate constitutes a principal component of the total inorganic carbonates under natural conditions (pH 6–9) [2]. Their solution chemistry, along with that of alkali and alkaline earth metals, is of paramount importance for aquatic life and also influences the distribution of flora and fauna [3].

Therefore, a fast, accurate, and reliable means of monitoring HCO_3_^−^ in such media will provide a key tool to ensure the proper functioning and understanding of carbonate equilibria. Moreover, it will serve as a safeguard against drastic changes in alkalinity as a consequence of different metal complexation processes [4]. Bearing that in mind, various instrumental methods have been developed so far [5]. However, it is important to remark that many of these approaches solely depend upon laboratory-based analysis due to a lack of techniques that can be utilized on-site, the use of ion selective electrodes (ISEs) [6] being one of the most important exceptions. Therefore, and owing to the vital impact of water soluble HCO_3_^−^ and the ever-better performance of these portable analytical means for “in situ” analyses, this potentiometric technique has become the cornerstone in the present research work.

As widely shown in the literature [7], the equilibrium concentrations of the different species (H_2_CO_3_/CO_2_, HCO_3_^−^, CO_3_^2−^) in an aqueous solution of bicarbonate ions depend on the pH value of the medium, in such a way that they can be calculated as a function of said value as well as the two dissociation constants of carbonic acid (K_1_ = 10^−6.4^ and K_2_ = 10^−10.3^). This is the procedure on which several ISEs are based to determine the bicarbonate concentration in all types of waters and aqueous solutions; it can be carried out (a) directly or (b) by measuring CO_2_ or carbonate [HCO_3_^−^], being then automatically calculated using the pH of the solution. In order to obtain this last value, the aforementioned ISE is equipped with a glass electrode that simultaneously determines the pH value.

Our research work aims at achieving the design and development of smart sensors for the upcoming Internet of Things (IoT). For example, the future in IoT describes a scenario where a set of environmental sensors will upload their measurements in a cloud service; in this way, it will be possible to know the concentrations of different species occurring in water, since they are recorded on a common IoT platform. For such an application, the aforementioned sensors must be provided with the ability to integrate themselves on IoT platforms [8].

In this paper, we propose a methodology for this integration, based on the so-called distributed rational agents (DRAs); they allow for achieving this goal, i.e., the design of sensors that benefit from cloud services offered by IoT. Examples include interference from polluting agents, influence of temperature and/or pressure, or other existing open data.

The utilization of this methodology will provide a bicarbonate smart sensor with the ability to integrate into IoT platforms, thus improving its performance, since it will be able to determine [HCO_3_^−^] without needing to include a pH sensor (pH data will be retrieved from the cloud). This leads to greater simplicity of the sensor, as well as lower cost and energy consumption.

On the other hand, a wider objective will be achieved. There is no doubt that this is a future trend in many fields, including electroanalytical chemistry. In this sense, these sensors will provide the cloud with suitable information, on the assumption that they are connected to the Internet [9]. At any rate, this is an initial approach to this issue, although it can be clearly perceived as an indicator for future developments over the short-term.

## 2. IoT and Cloud-Based Services in Analytical Chemistry

The evolution of computer systems, as well as the miniaturization of electronic devices and the improvement of communications, have allowed for the emergence of wireless sensor networks (WSNs), which measure and transmit data. WSNs are now a well-established technology [10].

Smart and distributed sensing systems are one of the technological cornerstones of the Internet of Things (IoT) [11], applicable to many fields, such as wearable sensors, industrial smart metering, and environmental monitoring. Today, one of the major challenges of using smart wireless sensors in real deployments is related to energy consumption, cost, and guaranteeing adequate lifetime. The support for the remote management (reception, storage, processing, and sharing) of these data is provided by so-called cloud computing technologies. They consists of the presence of Internet servers—clusters—which offer data carrier services through the Internet, including:

- SAAS (Software as a Service): it provides the end user with final applications of Internet-accessible data.
- PAAS (Platform as a Service): it allows for the development of applications that take advantage of the environment (ubiquity, high availability).
- IAAS (Infrastructure as a Service): it permits one to obtain computer resources in the form of virtual machines, which gives rise to seemingly endless scalability.

**Figure 1:**
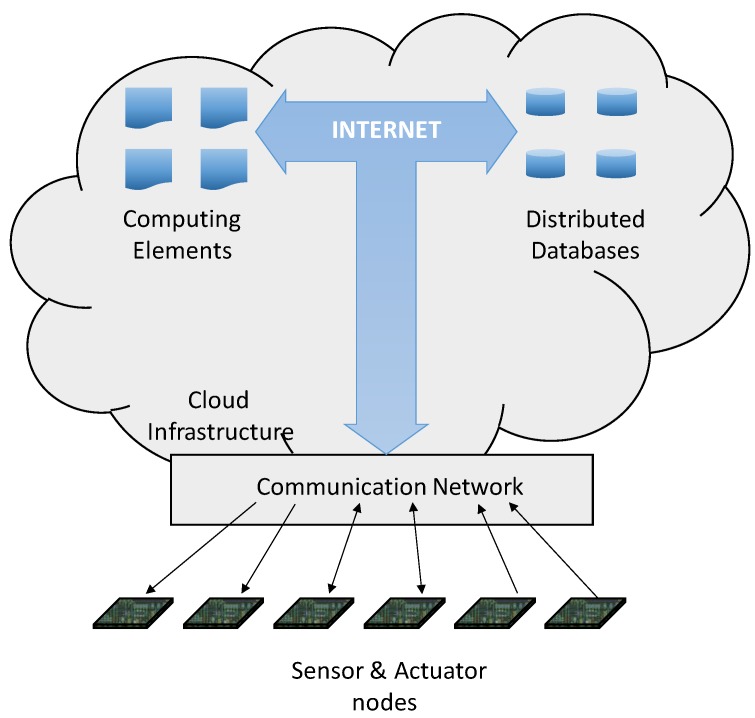
Different elements combined within an Internet of Things (IoT) cloud-based platform.

Figure 1 shows the different elements that are combined in a cloud-based IoT platform. Through the communication infrastructures, all sensors and actuators are able to exchange information with the cloud; sensors provide the information, whereas actuators carry out actions on the environment. This information can be stored on distributed databases that are utilized by several applications (running in the cloud) in order to provide an added value. In this sense, the data carrier provided by the cloud also includes their processing, i.e., the combination of different data to produce a new knowledge. Sometimes this is an easy process (for example, the search for conventional statistical information or basic operations), but it is not always so; in other cases, the great amount of data to be stored on these cloud systems require the application of Big Data techniques (the processing of a large volume of information in order to achieve added value). In this sense, Big Data allows for predictive analysis, trend searches, and data correctness assessment.

Given the increasing complexity of these systems, it is necessary to resort to some abstractions for an easier implementation in an intuitive, systematic, and structured way. For this purpose, the use of the so-called ‘intelligent agents’ is proposed as one of the most suitable paradigms. An agent (A) is anything that can be viewed as perceiving its environment through sensors and acting upon that environment through actuators. For each possible percept sequence, a rational agent (RA, also known as an intelligent agent) should select an action that maximizes its performance measure (in expectation) given the evidence provided by the percept sequence and whatever built in knowledge the agent has. Finally, a distributed rational agent (DRA) is an RA composed of different functionalities (consisting of hardware/software modules), which can be located in different network nodes, and all of them are connected by means of a communication network through which information is exchanged.

**Figure 2:**
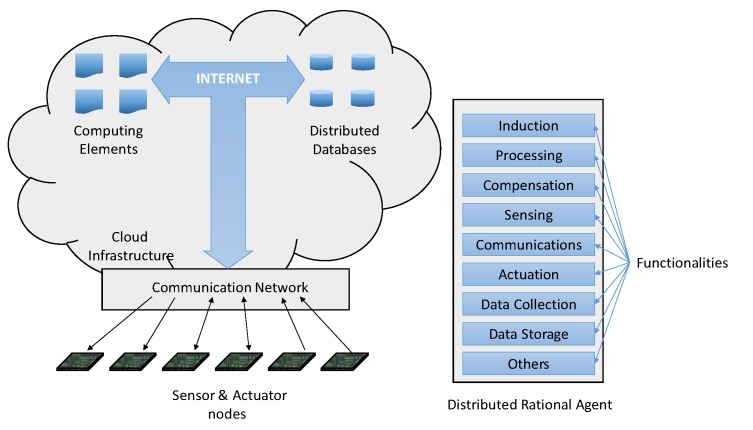
Integration of the distributed rational agents (DRAs) in an IoT cloud-based application.

Figure 2 summarizes the integration of a DRA into a cloud-based IoT environment. This DRA has been added to the scheme shown in Figure 1, in such a way that the different functionalities, according to specific optimization criteria, can be placed either in the sensors/actuators side or in the cloud side, or even in both of them. For example, if the calculation part requires a great amount of CPU resources, then it can be carried out in the cloud; in doing so, the consumption of the node is reduced, its lifetime being longer. Among the different options, two of them are noted: At one end, there can be very simple nodes that only transmit raw data to the cloud, all other processes being performed in the cloud to get the expected data. On the other side, very powerful nodes may be available so that all functionalities can be located in the node itself, expected data being sent to the cloud following the open-data philosophy.

The DRA indicated in Figure 2 does not show all possible functionalities, nor is it necessary that each sensor/actuator implements all of them. Depending on the application, the designer will select which ones are to be implemented and where.

The interaction of a huge number of objects, as well as their diversity, and the processing of all the available information opens up new prospects for society. An intelligent object will be able to process the self-generated data using additional information that can be obtained from other objects.

It is very significant, as shown in Figure 2, that IoT can integrate data from multiple sources and object categories. For instance, data required by a particular application may come from either private agents (firms and users who voluntarily upload this information) or public institutions (administrations, universities, research centers). In spite of data heterogeneity, the task of integration of all this information and the use of suitable IoT platforms permit their easier interoperability (although it is not guaranteed).

In the near future, trends show the possibility of obtaining nearly any measured data (regardless of the agent) on a common platform, and accessible for any application—be it public or private—that needs them. Initiatives such as open data from New York City or Valencia (our hometown in Spain) already offer a great amount of data, both from the public domain or provided by private agents.

One of the most promising features of IoT is the ability of the objects to integrate information processing routines, thus providing a faster local service; alternatively, it can resort to a more traditional cloud computing approach, according to which the computation is located in the cloud, like in the WSN.

**Figure 3:**
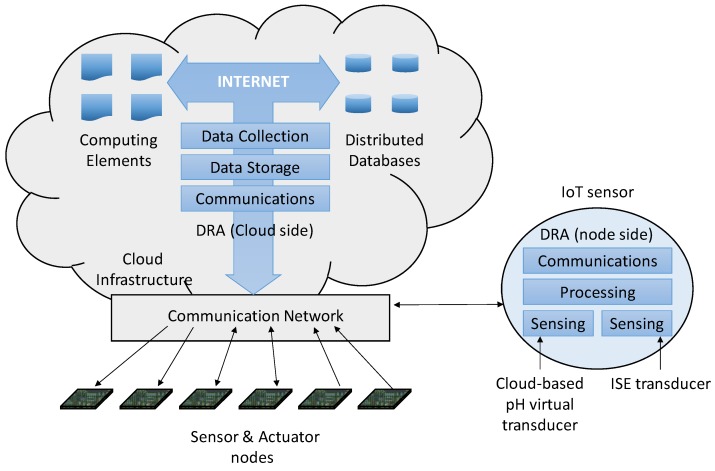
DRA distribution scheme in the proposed application.

Figure 3 shows how the node implements both sensorization and basic communications functionalities, whereas the access to the open database is remotely provided for obtaining and publishing information. There is an intermediate step between the two functionalities: the processing of the acquired information to get a processed measurement (shown in Figure 3 as a DRA).

Its main feature is the fact that it can be run on different hardware environments, depending on its requirements. In this environment, information processing may be carried out in two ways:

(a) Processing in the node: In this case, the smart sensor can directly access the public database in order to obtain the values it needs, and thereafter it is able to generate the final output. This mode has the advantage that the node offers greater intelligence, thus directly providing the processed information; this means that there is no need to transmit the obtained measurements (very abundant in some cases). They are also straightforward with data privacy and confidentiality, since both raw data and their processed values remain in the node itself. On the other hand, this mechanism needs a higher processing capacity in the node, and therefore, greater energy requirements.
(b) Processing in the Cloud: On a reciprocal basis, the node transmits the obtained measurements for their further processing in the cloud infrastructure. This means that the node does not directly interact with the public database, but there is an intermediate layer that is able to collect the corresponding data, as well as to receive the measured values and to process them in order to get the final result. Its most significant advantage consists of the reduction in both computing power and node consumption, even though a final transmission of the result is required if the node has to manage the processed information. The opposite case is a sensor network.

One aspect that should be underlined is the vast range of existing cloud services, which guarantees the service quality as well as the confidentiality and integrity of data and processes. In fact, they are straightforward with enhanced performances in terms of availability, with lower costs, since they take advantage of the benefits of economies of scale.

The application of IoT technologies leads to some troublesome issues, such as information storage and management, and ubiquitous access (while ensuring security and privacy). On the other hand, cloud computing environments provide storage and computation ubiquitous services that may address these needs. Therefore, the symbiosis between both ecosystems is highly profitable. An interesting example, in this case related to health issues, is shown in [12].

There is scarcely any doubt that analytical chemistry (as well as many other disciplines) will soon benefit from the important aforementioned advantages of cloud computing and IoT. Nevertheless, to date a detailed search in the literature has resulted in only a few relevant contributions [13,14,15,16,17,18,19,20,21,22,23,24], hence the importance of the present work as a somewhat pioneering contribution in this application field.

## 3. Application to the Determination of Bicarbonate in Water

The first step consisted of the incorporation of this paradigm into an application for the determination of bicarbonate concentration using carbon dioxide sensors. For this purpose, we needed to know the pH value, which was obtained from the cloud.

The determination of HCO_3_^−^ concentration can be carried out using an appropriate ISE [25]. Nevertheless, this is unusual, since the optimum pH working range is 7–8 and real sample values are generally lower, ISE response then being then unsatisfactory. Instead, in most cases carbon dioxide is measured by means of either a carbon dioxide ISE or a carbonate ISE (coupled with a glass electrode), and the concentration obtained is converted into [HCO_3_^–^] using the pH value provided by the glass electrode [26].

However, in some cases there are pH meters that are able to continuously monitor the pH values of an aqueous solution, the obtained measurements being gathered on open-access free public IoT platforms. In this regard, since the sensors must be equipped with communication mechanisms to provide the database with this information, it could be considered that glass electrodes in the ISEs are not essential; in fact, they raise the overall costs as well as energy consumption (and the information provided would be redundant). Furthermore, we subscribe to the pH information, so that [CO_2_] is measured when the provider updates the pH values. Nevertheless, if the sensor detects that there has been no updating in the last 12 h, [CO_2_] is measured using the latest available pH value despite of the low reliability. Our proposal in this paper takes into account the ever-increasing number of data providers (existing WSN, user contributions, results from other IoT processes) and the appearance of IoT-based integration platforms (they provide access to large open databases). On that basis, this new approach consists of the design and development of a simplified bicarbonate sensor based on a carbon dioxide ISE with IoT abilities. The main goal is to get pH values from open databases for the calculation of bicarbonate concentration.

The starting point of the methodology proposed is the sensor described by our research group [10], which consists of a microprocessor-based system equipped with a power supply that is recharged by means of a small solar panel. The core of the system consists of a low-consumption, low-cost microcontroller; its capacity is nevertheless more than sufficient to carry out all the operations required to apply the developed techniques. This microcontroller is a small integrated circuit that contains all the computer components (CPU, memory, and necessary I/O subsystems) and therefore offers the possibility to implement complete applications using only one chip. The device chosen (ARM Cortex M0) is a 32-bit microcontroller with high energy efficiency (12.5 μW/MHz) and performance; it has 2 KB RAM memory and 8 KB flash memory, as well as three timers (16 and 32 bits) and an A/D converter (10-bit resolution and 8 channels). Incoming signals from ISEs are adapted/amplified by means of an AD524 Instrumentation Amplifier (Analog Devices). Furthermore, it includes a CC1110 controller, which allows for its connection with a gateway to the Internet using the 868 MHz band. This node design was used again, it being fitted with the ISEs described in Section 4.

The following DRA functionalities were then incorporated into the sensor node shown in Figure 3:

- Processing Module: It is in charge of processing the data provided by the sensing functionalities. It also transmits the obtained measurement to the data storage module (located in the cloud).
- Sensor Modules: Two sensor modules were utilized in this application: The first one receives the data from the ISE, whereas the second one is a virtual pH transducer sensing functionality that gets the data from the data collection functionality (also situated in the cloud). Said module can be considered as a virtual transducer, since it does not obtain the data from a physical sensor (in the node) but from other agents that obviously have the corresponding physical transducers located in the cloud.

On the other hand, other DRA functionalities can also be found in the cloud. They include:

- Data Storage: This module receives the correct values measured by the smart sensor and stores them on the distributed database system, more specifically on the selected IoT platforms. These data become available to the rest of agents in the cloud; they may use them in the same way as the existing pH data.
- Data Collection: This module is in charge of getting the requested information from the cloud (pH in our case) by interacting with the other agents (platforms). This information is then transmitted to the sensor functionality, which interprets it and passes it on to the processing module.

## 4. Experimental

### 4.1. FiWare Infrastructure

FiWare [27] is an open architecture that allows one to build a sustainable ecosystem around public software platform standards, free of rights and based on implementation in order to facilitate the development of new smart applications in many sectors. This open source platform was selected owing to its robustness and reliability, as well as its great number of users. The implementation cost may therefore be considered as very low in comparison with other options. FiWare manages the concept of generic enabler (GE), which is related to a library of general purpose services that cover common functionalities in fields such as security, storage, cloud, data context, and Internet of Things. These services are available through usable application programming interfaces (APIs) so that developers can use those functionalities when implementing their own applications. Each GE can be considered a block that contains a set of APIs for the construction of smart applications. These GE are divided into seven technical chapters, which cover cloud hosting, data and context management, interfaces to networks and devices, advanced web-based user interfaces, security, Internet of Things, and finally applications/services and data delivery.

FiWare infrastructure was utilized for the implementation of the experiments that were carried out. Two applications were implemented with JavaScript on the FiLab platform. One of them captures the current pH value using a pH-meter connected to a Raspberry Pi, the value being periodically uploaded by means of message queue telemetry transport (MQTT, an open standard network protocol for publish–subscribe services between devices) messages. The other one, implemented on the aforementioned microcontrollers, is subscribed to this information by means of the context broker (Orion), so as to obtain the bicarbonate concentration from [CO_2_]. This information was accessed by the proposed smart sensor in order to calculate [HCO_3_^−^].

The ISEs described in Section 4.2, Section 4.3, Section 4.4, Section 4.5 and Section 4.6 were utilized.

### 4.2. ISE 1: Bicarbonate ISE (Direct Determination)

A homemade electrode was fabricated following to some extent the process outlined in [26]. This ISE consisted of a polyvinyl chloride tube covered with a thin (10–25 µm) HCO_3_^−^ selective membrane made from a mixture containing polyvinyl chloride, di-(2-ethylhexyl) sebacate, trioctyl tin chloride and an H^+^ interference-removing trifluroacetophenone (trifluoroacetyldecyl-benzene), a liquid solution containing 50 mM phosphate buffer and 0.01 M sodium chloride in the tube, and a lead wire connected to a Ag/AgCl reference electrode positioned in the tube. This electrode had the following features: a Nernstian slope of 55 ± 5 mV per decade change in activity (an expected Nernstian monovalent response), a limit of detection of 1.4 mg L^−1^ of bicarbonate, and a response time of less than 20 s. On the other hand, the drift of the electrode contacting a 10 mM bicarbonate solution was less than 0.5 mV·h^−1^, measured at constant temperature and with the electrodes continually immersed in the solution.

The electrode was conditioned in 0.1 M NaHCO_3_ for 24 h after one week of measurements. Sodium bicarbonate standard solutions were also utilized for calibrating the ISE.

### 4.3. ISE 2: Commercial Carbon Dioxide ISE (Classical Version)

The carbon dioxide ion selective electrode (9502BNWP) was purchased from Thermo Fisher Scientific (Waltham, MA, USA). CO_2_ measured was related to bicarbonate concentration using the pH values provided by the glass electrode.

This ISE had the following specifications: a Nernstian slope of 28 ± 5 mV per decade change in activity (an expected Nernstian divalent response), a limit of detection of 6.4 mg·L^−1^ of bicarbonate, and a response time of less than 15 s. On the other hand, the drift of the electrode contacting the 10 mM bicarbonate solution was less than 0.5 mV·h^−1^, measured at constant temperature and with the electrodes continually immersed in the solution.

### 4.4. ISE 3: Non-Severinghaus Carbon Dioxide ISE (Most Recent Version)

The electrode was prepared and assembled following the procedure described in [28], with only some small modifications. The presence of a glass pH electrode with combined Ag/AgCl reference (Ecotrode Plus) and a double junction Ag/AgCl reference electrode allowed for the conversion of the CO_2_ measured into bicarbonate concentration electrode.

This electrode then had the following characteristics: a Nernstian slope of 27 ± 5 mV per decade change in activity (an expected Nernstian divalent response), a limit of detection of 5.3 mg·L^−1^ of bicarbonate, and a response time of 5 s. On the other hand, the drift of the electrode contacting the 10 mM bicarbonate solution was less than 0.5 mV·h^−1^, measured at constant temperature and with the electrodes continually immersed in the solution.

ISE 2 and ISE 3 are considered to be very similar, since ISE 3 is really an enhanced version of ISE 2. Owing to its improved performance, we believe that there is no need to further compare ISE 2 with the other devices, since ISE 3 better represents the classical determination of CO_2_.

### 4.5. ISE 4: Proposed Sensor

A homemade conventional CO_2_-ISE was modified accordingly, and later connected to the node described in Section 3. Then, this ‘virtual’ ISE provided [CO_2_] data and [HCO_3_^−^] values were calculated therefrom using the corresponding equations and pH data retrieved from the cloud. As mentioned above, these calculations could be carried out either in the node or in the cloud, the former being what was done in our case; pH was obtained from the FiWare server (located in the cloud), the final result was then displayed in the node, and [HCO_3_^−^] was finally sent to the cloud for its storage.

This electrode had then the following features: a Nernstian slope of 27 ± 5 mV per decade change in activity (an expected Nernstian divalent response), a limit of detection of 6.0 mg·L^−1^ of bicarbonate, and a response time of less than 20 s. On the other hand, the drift of the electrode contacting the 10 mM bicarbonate solution was less than 0.5 mV·h^−1^, measured at constant temperature and with the electrodes continually immersed in the solution.

### 4.6. Experimental Details

All solutions were maintained, using a thermostat, at 25 °C. This temperature was chosen because the values of acid–base dissociation constants listed in most of the tables are referred to at 25 °C.

All solvents and reagents used were analytically pure. Sets of 10 aqueous bicarbonate solutions (concentrations ranging between 30 and 300 mg·L^−1^) were prepared by dissolving the appropriate amounts of NaHCO_3_ in Milli-Q purified water. The pH values of these solutions were adjusted in a range between 4.5 and 7.0 using the corresponding buffers. ISE 1 directly measured bicarbonate concentration, whereas ISE 2 and ISE 3 evaluated the levels of CO_2_ and converted them into bicarbonate concentrations using the pH measured. Finally, ISE 4 also measured CO_2_, but the values obtained were transformed into bicarbonate concentrations from pH data contained in the cloud.

The pH values were monitored potentiometrically every six hours and simultaneously entered into the cloud database. They were obtained at the same time as the bicarbonate measurements, all of them being the mean value of *n* replicates (see the end of this section).

All four sensors were deployed nonstop for 24 h, calibration checks being performed twice a day; they showed a negligible drift during that period of time.

In order to obtain reliable results, *n* replications were performed for each measurement, *n* being calculated as follows: 

The results for each measurement were considered as random variables (*X_1_, X_2_, …, X_n_*) with a μ mean value; *n* measurements were repeated until an estimation of μ was obtained with a 90% confidence interval according to the expression:

$$\bar{X}\left(n\right)\pm t_{n-1,0.95}\sqrt{\frac{S^{2}\left(n\right)}{n}}$$

where *t_n –_*_1, 0.95_ represents the upper limit of the Student’s t-distribution on *n* – 1 degrees of freedom, and *X*(*n*) and *S*^2^(*n*) are the mean and the variance of the results obtained in the different experiments. In general, 5–15 replications were carried out for each measurement.

## 5. Results and Discussion

**Figure 4:**
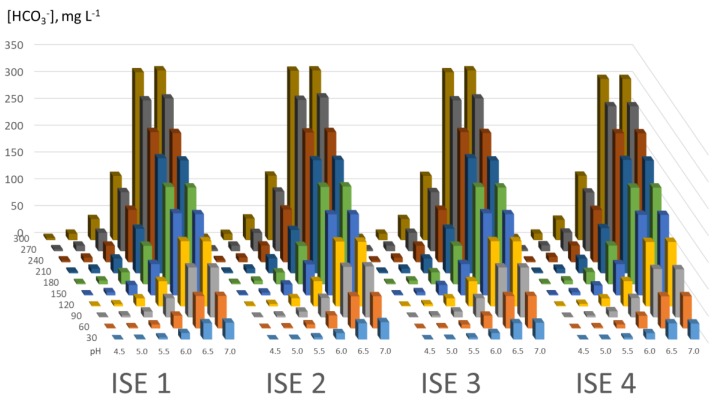
Comparison of the results obtained with the four ion selective electrodes (ISEs).

The experimental results obtained are shown in the following graphs (Figure 4).

As stated in Section 4.4, the comparison between ISE 2 and ISE 3 does not seem relevant to us, and therefore ISE 3 is considered to be representative of commercial CO_2_ ISEs. That is why no comparison between ISE 2 and ISE 3 was carried out.

In order to compare the results obtained from ISE 1 (direct measurements; assumed to be the most accurate value) with those from ISE 4, relative errors (RE) were calculated using the following expression:

RE = (value ISE 4 – value ISE 1)/value ISE 1.

The same procedure was utilized in the case of ISE 3:

RE = (value ISE 3 – value ISE 1)/value ISE 1.

**Figure 5:**
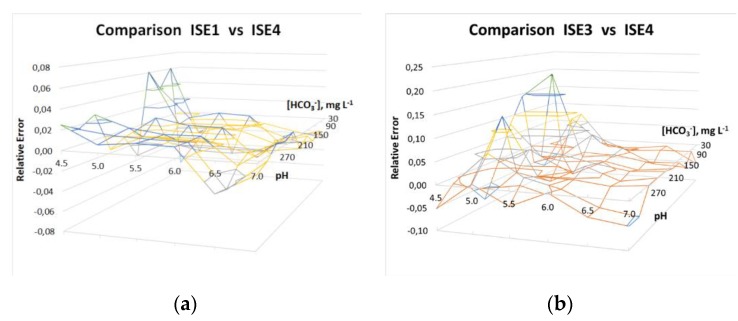
Errors in the results obtained: (**a**) ISE 1 vs. ISE 4, (**b**) ISE 3 vs. ISE 4.

The results obtained in both comparisons are summarized in Figure 5a,b.

As shown in Figure 5a (ISE 1 vs. ISE 4), the error was lower than 2% in most cases, and did not exceed 3% in 90% of cases. As expected, Figure 5a shows that the maximum relative error took place when both pH and the magnitude measured were very low. This case corresponded to a 6.9% relative error. Root Mean Square Error (RMSE) was also calculated, a value of 0.021 being obtained.

When the comparison was made with regard to ISE 3 (Figure 5b), accuracy was not so high, but it could be observed that more than half of all data showed an error under 2%, two-thirds of them being lower than 3%. In this case, the errors in pH and [CO_2_] measurements were accumulated, the highest relative error being then 20% (unacceptable, but corresponding to a very uncommon situation). On the other hand, for common pH values of water (5.5–6.5), the maximum error was lower than 4%. Even in the rare instance of pH = 7, the error was still low for bicarbonate concentrations between 120 and 240 mg·L^−1^. As in the previous comparison, RMSE was also calculated, a value of 0.049 being obtained.

As regards response times, those of the ISEs studied were in the range between 5 and 20 s. On the other hand, access time for cloud data was estimated to be 1−2 s, whereas by calculation it turned out to be negligible (a few milliseconds), which means that the response time of ISE 4 was limited by the acquisition capabilities of the CO_2_ ISE. Furthermore, while the ISEs utilized in these experiments had a response time of less than 20 s, state-of-the-art technologies (such as those used in ISE 3) could reduce it to 5 s, with a lower energy consumption (no pH electrode).

When dealing with energy consumption, ISE 4 operation was monitored in order to evaluate its behavior. It was found that ISE 4 required about half the energy of ISE 3 for correct operation.

## 6. Conclusions

A new approach was presented for the development of sensors based on cloud services and the Internet of Things. Following this methodology, and as an example, a new smart sensor was designed and tested; it is a bicarbonate smart sensor (cheaper, simpler, and more efficient) based on a combination of a real CO_2_ ISE with a virtual pH-meter transducer, which obtains its values from a cloud service that supports the IoT platform used.

The starting point was a study that clearly shows the existing symbiosis between analytical chemistry and the new technologies such as IoT and cloud-based services. In this way, the former can benefit from the advances provided by the latter; conversely, these state-of-the-art technologies need new sensors with special features that should be offered by new developments in analytical chemistry.

With a view to facilitating the design of these new sensors, the present work proposes a new approach based on the application of intelligent agents (DRA, distributed rational agents), whose functionalities may be implemented either in the cloud or in the sensor and actuator nodes themselves. According to the application and the sensor type, it is necessary to define the functionalities to be implemented as well as their location.

As an example of application of the proposed methodology, a new sensor for the potentiometric determination of bicarbonate in water is described. A carbon dioxide ISE is utilized for this purpose, but with an important novelty: The ISE measures the concentration of CO_2_ and, using the pH data available in the cloud, is able to provide the values of bicarbonate concentrations. Moreover, the overall energy consumption is substantially lower, since no additional electrode is required for measuring pH. This also leads to a decrease in both cost and size. When it comes to the implementation of this sensor, DRA functionalities are distributed between the cloud and the sensor node in such a way that sensing and processing attributions are located in the sensor node, whereas data collection and data storage remain in the cloud. Communication modules between both blocks are also provided.

From a chemical point of view, the results obtained are quite encouraging. Taking into account the most common pH values of water (in the range of 5.5 to 6.5), satisfactory accuracy and precision were obtained (as shown in the experiments of comparison with the other sensors). On the other hand, no significant operation errors were detected and there were only a few data transmission errors, recovered by the communication functionalities using common retransmission protocols. However, in any case, it must be remarked that this is an initial investigation, and therefore some work still needs to be carried out to achieve the ultimate goal of developing a truly smart sensor using IoT and cloud services.

Additionally, with this proposal it is possible to infer indirect determinations based on easier and inexpensive measurements, along with the information available on existing databases, both public and private. As shown in the previous sections, the information of these IoT platforms can be utilized for specific applications, thanks to the disconnection between sources and consumers. In this way, it is possible to develop sensors that can ‘virtually’ determine (and upload to the IoT platform) some chemical parameters without physical transducers. Finally, a virtuous cycle takes place in this environment: The more public information available, the greater the number of benefitted applications, which also means more available data.

In conclusion, the most relevant contribution of this work consists of the application of IoT and cloud-based services in analytical chemistry environments, such as the potentiometric determinations of ionic species in aqueous solutions. This new ecosystem is based on the confluence —on a single exchange platform—of data from multiple sources (sensor networks, industrial systems, personal contributions, and so on) that can be employed by any user. Following this line of thinking, the amount of information of this kind available in the cloud is growing exponentially. Therefore, it is necessary to implement the integration of analytical chemistry into the new IoT applications (as proposed in this paper). Undoubtedly, further advances in this line will open up a whole new and exciting world of IoT applications.
